# Osteoinduction of Human Mesenchymal Stem Cells by Bioactive Composite Scaffolds without Supplemental Osteogenic Growth Factors

**DOI:** 10.1371/journal.pone.0026211

**Published:** 2011-10-12

**Authors:** Alessandro Polini, Dario Pisignano, Manuela Parodi, Rodolfo Quarto, Silvia Scaglione

**Affiliations:** 1 CNR - National Research Council of Italy, NNL (National Nanotechnology Laboratory) of Institute Nanoscience, Lecce, Italy; 2 Dipartimento di Ingegneria dell'Innovazione, Università del Salento, Lecce, Italy; 3 Dipartimento di Medicina Sperimentale (DIMES), Università di Genova, Genova, Italy; 4 CNR - National Research Council of Italy, IEIIT Institute, Genova, Italy; Pennington Biomedical Research Center, United States of America

## Abstract

The development of a new family of implantable bioinspired materials is a focal point of bone tissue engineering. Implant surfaces that better mimic the natural bone extracellular matrix, a naturally nano-composite tissue, can stimulate stem cell differentiation towards osteogenic lineages in the absence of specific chemical treatments. Herein we describe a bioactive composite nanofibrous scaffold, composed of poly-caprolactone (PCL) and nano-sized hydroxyapatite (HA) or beta-tricalcium phosphate (TCP), which was able to support the growth of human bone marrow mesenchymal stem cells (hMSCs) and guide their osteogenic differentiation at the same time. Morphological and physical/chemical investigations were carried out by scanning, transmission electron microscopy, Fourier-transform infrared (FTIR) spectroscopy, mechanical and wettability analysis. Upon culturing hMSCs on composite nanofibers, we found that the incorporation of either HA or TCP into the PCL nanofibers did not affect cell viability, meanwhile the presence of the mineral phase increases the activity of alkaline phosphatase (ALP), an early marker of bone formation, and mRNA expression levels of osteoblast-related genes, such as the Runt-related transcription factor 2 (Runx-2) and bone sialoprotein (BSP), in total absence of osteogenic supplements. These results suggest that both the nanofibrous structure and the chemical composition of the scaffolds play a role in regulating the osteogenic differentiation of hMSCs.

## Introduction

Regenerative medicine aims to repair and replace lost or damaged tissues by initiating the natural regeneration process. Current paradigms in tissue engineering often involve the combination of mesenchymal stem/progenitor cells and the synthesis of novel biomaterials, tailoring physical, chemical and structural properties to mimic crucial aspects of the physiological niche [1]. Ideally, the scaffold design is aimed at reproducing all required signals at macro, micro- to nano-scales, respectively corresponding to tissue, cellular, and molecular scales in a specific tissue, in order to foster and direct cellular attachment, proliferation, desired differentiation towards specific cell phenotypes. In this context, several factors must be considered, such as the chemical nature of scaffolding material, the physical structures at various size scales, and the method of fabrication [2]–[6].

Several studies have indicated that the presence of a mineral biomimetic phase, such as hydroxyapatite (HA) or tricalcium phosphates (TCP), is important for the success of a scaffold promoting bone regeneration [7], [8]. HA is more stable, exhibiting much lower dissolution rates, whereas beta-TCP is more soluble and its degradation products, Ca^2+^ and PO_4_^3−^, are released into the surrounding environment, potentially inducing bioactivity [8], [9]. However, the use of either compound is hampered by difficulties in processing into highly porous structures and native brittleness. In contrast, many synthetic biodegradable polymers are proposed in various tissue engineering applications, including bone tissue repair, based on their flexibility of material properties and the ability to support cell growth [2], [10], [11], but they typically lack in osteoconductive properties. A good compromise may be represented by composites joining polymer plasticity with the osteoinductivity of phosphate ceramics.

Besides the chemical composition, also the micro-nano-structural properties of the bone substitutes have to be accurately defined, since the surface morphology, stiffness or topography of scaffolds can directly and significantly affect cell-scaffold interactions and ultimately tissue formation [3], [5], [12]–[13]. To date, a very few studies report on hMSCs differentiated in vitro into osteogenic [14]–[16], neuronal [17], or muscular [18] lineages without any exogenous soluble differentiation factor, exploiting predetermined micropatterns and geometries. Following a biomimetic approach, the use of nanofibers structures would add further value in this framework, by mimicking the intricate fibrillar architecture of natural extracellular matrix (ECM) components. In fact, the ECM plays an important role in regulating aspects of cell division, adhesion, cell motility, differentiation and migration, modulating growth factors distribution, activation, and presentation to cells [19], [20]. Therefore, the development of an artificial ECM, performing the structural and biochemical functions of native ECM, represents a promising approach. Polymer processing technologies, such as electrospinning, allow the production of scaffolds with a morphology characterized by a nanofibrillar structure and have been successfully employed for tissue engineering applications [21]. Previous studies focusing on electrospun scaffolds for bone tissue engineering, have employed a wide range of materials for inducing bone differentiation, but always using osteogenic medium [22]–[37]. The effects related to the use of basal or osteogenic media are rarely reported for Polycaprolactone (PCL) scaffolds [36], [37], showing no evidence of intrinsic osteoinductive properties for the scaffold (i.e. in the absence of osteogenic supplements).

This work describes biomimetic, bioactive composite scaffolds and their ability to induce hMSCs towards an osteogenic differentiation, exploiting their chemical and nano-micro topological structure. PCL, one of the most popular synthetic polymers approved by the US Food and Drug Administration [31]–[37], was used as polymer matrix , while HA and TCP represented the mineral phase. Our findings highlight a mature osteogenic differentiation of hMSCs when cultured in vitro in basal growth medium conditions onto nanofibrous ceramic-polymer materials. An increased Alkaline Phosphatase (ALP) activity and mRNA expression modulation of the most typical osteoblast-related genes were observed.

## Materials and Methods

### Scaffolds fabrication

PCL powders (Mw 70,000–90,000, Sigma-Aldrich, St. Louis, MO) was dissolved in hexafluoroisopropanol (HFIP, Carlo Erba, Milan, Italy) under stirring for a few hours, obtaining a 3.5% concentration. For composite fibers, HA (average size 20–70 nm) or beta-TCP (average size 100 nm) nanocrystals (Berkeley Advanced Biomaterials, San Leandro, CA) were added to the PCL/HFIP solution and put under stirring for one week, using oleic acid (0.05% w/v) as surfactant in order to obtain stable particle suspension in the polymer solution [32]. Three different solutions were prepared: 3.5% PCL, 3.5%–2% PCL-HA, 3.5%–2% PCL-TCP using HFIP as solvent (all the concentrations are expressed as w/w ratios in relation to the solution). For the electrospinning process, each polymer solution was loaded in a plastic syringe with a 27 gauge stainless steel needle, and a 4.5 kV voltage was applied using a high voltage power supply (EL60R0.6–22, Glassman High Voltage, High Bridge, NJ). The injection flow rate was fixed at 0.5 mL/h, supplied by a microfluidic syringe pump (Harvard Apparatus, Holliston, MA). The nanofibers (NFs) were collected on round (diameter 15 mm) or rectangular (75×25 mm^2^) borosilicate glass coverslips, mounted on a grounded 10×10 cm^2^ collector at a distance of 20 cm from the needle, for 2 hs. The air relative humidity and temperature conditions were about 40% and 23°C, respectively. As control, the same electrospinning solutions were employed for obtaining film samples by spin coating (5000 rpm for 60 sec). All the samples were stored in a vacuum desiccator at room temperature for at least one week, to remove any residual HFIP molecule. At the end of the procedure, six different scaffolds were available: PCL, PCL-HA and PCL-TCP, both in the nanofibrous structure and film. All samples were sterilized by gamma-irradiation (5000 rad) before cell culture.

### Scaffolds characterization

The morphology of nanofibrous samples was analyzed by electron microscopy. For scanning electron microscopy (SEM), NFs were imaged by a Raith 150 system (Raith, Dortmund, Germany) using an accelerating voltage of 5 kV and an aperture size of 20 µm. No metal was deposited on samples before SEM, for better evidencing embedded nanocrystals. The average fiber diameter was calculated analyzing at least 100 NFs by an imaging software (WSxM, Nanotec Electronica, Madrid, Spain) from various binarized SEM images. Briefly, for each image several lines, perpendicular to the longitudinal axis of each fiber, were traced and the diameter was calculated from the resulting signal intensity vs. position plot. Transmission electron microscopy (TEM) pictures were obtained using a Jeol Jem 1011 microscope (JEOL, Tokyo, Japan) operating at an accelerating voltage of 100 kV. The samples were prepared by directly electrospinning on TEM carbon filmed copper grid with 300 mesh (TAAB Laboratories Equipment, Aldermaston, England), mounted on the collector. The presence of nanoparticles and the resulting chemical composition of NFs mats were analyzed by Fourier-transform infrared (FTIR) spectroscopy (Spectrum 100, Perkin Elmer, Waltham, MA) in transmission mode. Spectra were recorded in the 450–4000 cm^−1^ range with 256 scans at spectral resolution of 2 cm^−1^, averaged and baseline-corrected. A dynamic mechanical analyzer (DMA Q800, TA Instruments, New Castle, DE) was employed to perform tensile measurements on the nanofibrous mats. Each sample (n = 5 specimens) was cut into rectangular shapes (6 mm×20 mm), measuring the thickness with a digital micrometer prior to testing, and subjected to a ramp/rate of 1 N/min (up to 18 N). Water contact angle analysis (CAM-200 KSV Instruments, Helsinki, Finland) was performed dropping deionized water (2 µl) from a syringe onto the surface of each sample (n = 5 specimens).

### Cell culture

hMSCs were purchased by Lonza (Milan, Italy). A pool of 5 healthy donors was resuspended in Mesenchymal Stem Cell Growth Medium (MSCGM, Lonza) and cultured in humidified 5% CO_2_, 37°C incubator, removing non adherent cells after 48 h of incubation. Cells were expanded in vitro until passage 1, and then seeded onto the nanofibrous and film scaffolds at sub-confluence density (45000 cells/cm^2^). Cell culture on plastic surface was used as control. Cells were cultured both under basal (BM) and osteogenic (OM) conditions for two weeks. Osteogenic medium was purchased by Lonza (Differentiation BulletKit). At day 7 of culture, some nanofibrous and film scaffolds, cultured in BM, were processed for SEM. Samples were washed in sodium cacodylate buffer for 10 min, fixed in 2.5% glutaraldehyde buffer for 30 min at 4°C, and rinsed twice in cacodylate buffer solution. Scaffolds were then dehydrated in increasing concentrations of ethanol, air dried, sputtered with a nano-gold film and analyzed by SEM (CrossBeam 1540XB, Carl Zeiss, Oberkochen, Germany). Cell proliferation assay was performed according to AlamarBlue (Invitrogen, Milan, Italy) instructions, every 24 hours for a week, in proliferation medium (BM).

### ALP activity

ALP enzyme activity of hMSCs, either cultured on NFs or control film, was assessed after 7 days of cell culture in either basal or osteogenic medium. ALP staining was performed according to manufacturer's instruction (Sigma kit 86-R, Sigma-Aldrich). Cell culture on plastic surface was used as control. After staining, samples were digitally photographed, and acquired images were analyzed using an open source image analysis software (Image J, NIH). The images were converted in gray-scale (0–255 bit), a region of interest (ROI) of 41516 px was selected, the mean intensity value and its standard deviation were determined for each image.

### RNA extraction and RT-PCR

mRNA was isolated after 1–2 weeks of culture on plastic or our materials, either under BM or OM, by using RNAeasy micro kit (Qiagen, Milan, Italy). Sample amount was determined by spectrophotometric quantification. All mRNA samples were treated with deoxyribonuclease I (Invitrogen) prior to reverse transcription. First strand cDNA synthesis was performed using equal amount of RNA samples (2 µl), according to M-MLV RT instructions (Invitrogen). Gene expression levels of core binding factor alpha 1 (CBFA1/RUNX-2), collagen type 1 (Col-I) and bone sialoprotein (BSP) were analyzed by real time PCR, using the Eppendorf Mastercycler ep Realplex2 (Eppendorf, Hamburg, Germany). Glyceraldehyde 3-phosphate deydrogenase (GAPDH) was employed as housekeeping gene. Primer sequences and annealing temperature are reported in Table S1. PCR reactions were performed using RealMasterMix SYBR Green (5prime, Hamburg, Germany) in a total volume of 13 µl. Each sample was assessed at least in duplicate.

### Statistical Analysis

For mechanical properties, water contact angle analysis and ALP staining semi-quantification, a one-way analysis of variance (ANOVA) with Tukey's post hoc test for multiple comparisons was used for statistical analysis (Sigmaplot 12.0, Systat Software Inc., Point Richmond, CA), and a P value <0.05 was considered statistically significant. Quantitative data on gene expression were obtained as the mean and standard deviation of values derived from three independent experiments carried out using cells from pulled donors. Differences were statistically assessed using Mann-Whitney non-parametric U-test, and considered statistically significant with P value <0.05.

## Results and Discussion

### Scaffolds characterization

**Figure 1:**
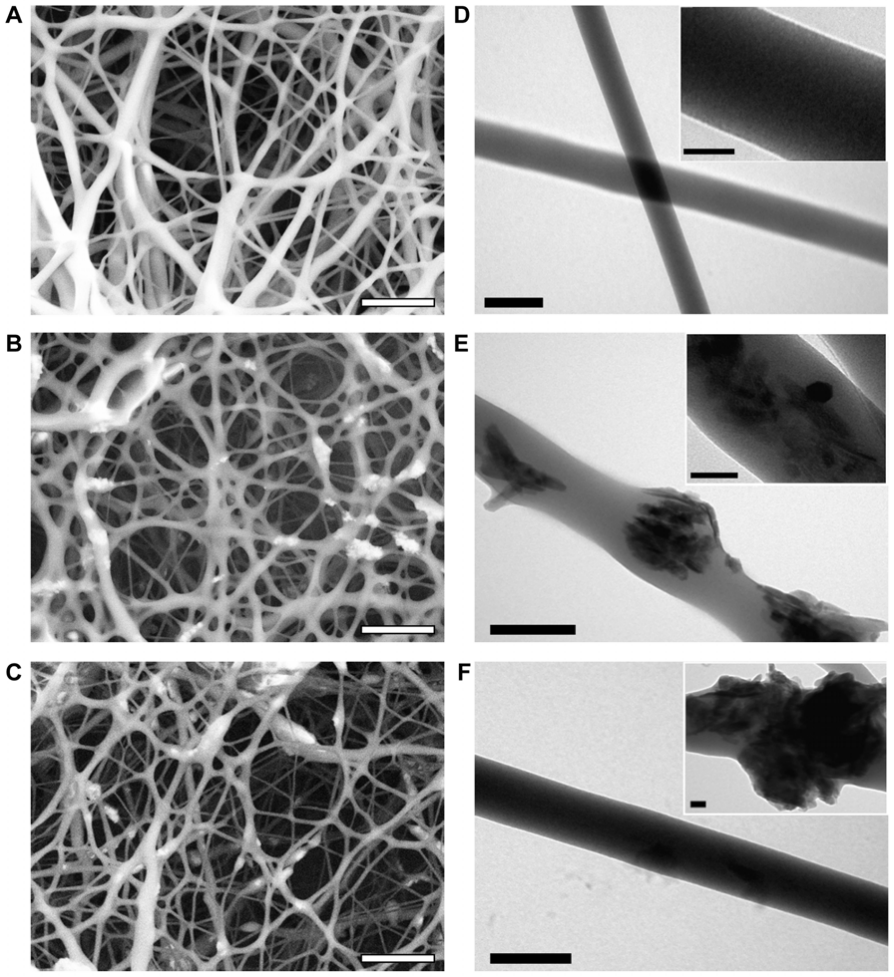
Electron microscopy investigation of electrospun nanofibrous scaffolds. SEM (A–C) and TEM (D–F) micrographs for PCL (A,D), PCL-HA (B,E) and PCL-TCP (C,F). Bar: 2 µm (A–C), 200 nm (D–F), or 50 nm (insets D–F).

In this study, we demonstrate that a composite nanofibrous scaffold alone can induce a significant modification of osteogenic markers, suggesting the induction into osteogenic lineage, of hMSCs. To evaluate this effect, we fabricated PCL, PCL-HA and PCL-TCP NFs using the electrospinning technique. In particular, the average fiber diameter was (210±100) nm, (230±130) nm, (225±100) nm, for PCL, PCL-HA, and PCL-TCP scaffolds (Fig. 1A–C), respectively, falling within the range of dimension characteristic of the ECM architectures [21]. Higher concentrations of nanoparticles in the composite solution unavoidably led to beads formation due to particles agglomeration, as noted using a 3.5% PCL 4% HA (or TCP) solution (data not shown).

TEM analysis confirmed the surface morphology of both PCL and PCL-composite fibers (Fig. 1D–F), with the latter displaying a protruded granulate-like morphology, also observed for HA-chitosan fibers [28]. Ceramic nanocrystals were present on the fiber surface as well as embedded in depth, allowing in principle a slow and constantly growing exposure of ceramic surface to the cells, while the polymer is degraded. This non-uniform distribution can be ascribed to the original dispersion, containing both well-dispersed particles and particle aggregates due to the high viscosity of the electrospinning solution [27]. While other studies have been focused on achieving composite fibers by preferentially orienting the particles parallel to the longitudinal axis of the fibers during the production [29], or by mineralizing the inorganic phase onto the organic phase after the realization [30], here the nanoparticles distribution makes the inorganic phase potentially available for the cellular microenvironment in a continuous and consistent way.

**Figure 2:**
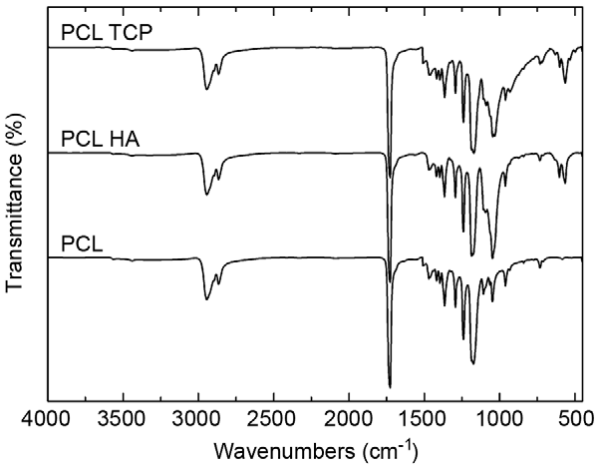
FTIR spectra of electrospun NFs. Typical infrared bands for PCL-related stretching modes were observed for both the pure polymeric and composite scaffolds: peaks at 2949 cm^−1^ (asymmetric CH_2_ stretching), 2865 cm^−1^ (symmetric CH_2_ stretching), 1727 cm^−1^ (carbonyl stretching), 1293 cm^−1^ (C–O and C–C stretching in the crystalline phase) and 1240 cm^−1^ (asymmetric COC stretching). For composite samples characteristic PO_4_^3−^ absorption bands attributed to HA or TCP nanoparticles were observed at 564 cm^−1^, 603 cm^−1^, and 1031 cm^−1^.

The chemical composition of the fiber mats, investigated by FTIR spectroscopy, confirmed the presence of nanoparticles within the scaffolds. Typical infrared bands for PCL-related stretching modes were notable for both pure polymer and composite scaffolds, with the latter showing characteristic PO_4_^3−^ absorption bands at 564, 603 and 1031 cm^−1^ addressable to HA or TCP nanoparticles (Fig. 2). These peaks were slightly shifted in respect to standard values for phosphate adsorption bands, which can be due to the interaction between the ceramic phase with the polymer phase [33].

**Figure 3:**
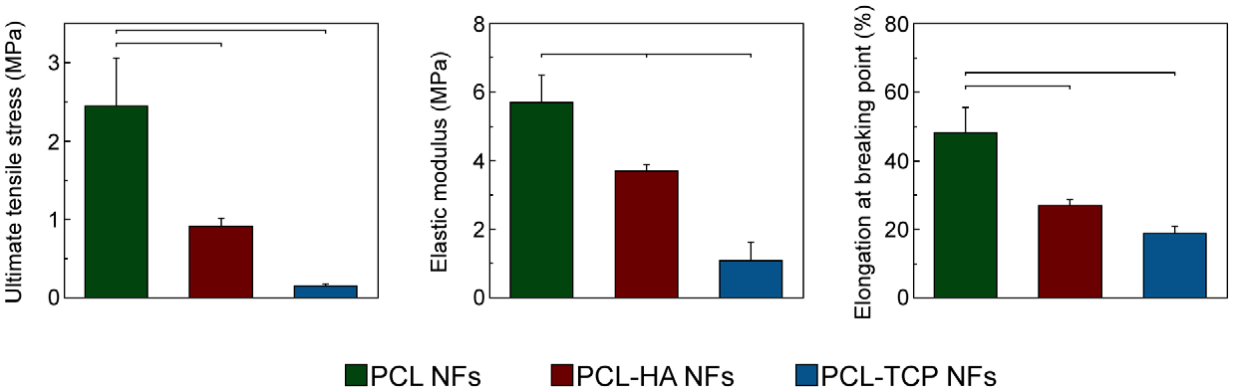
Mechanical properties of pure polymeric and composite nanofibrous scaffolds. Results are expressed as mean ± standard deviation. Bars show statistically significant differences (P<0.05).

The presence of nanoparticles embedded into PCL matrix affected the mechanical properties of the fiber nets (Fig. 3). While the elastic modulus values for polymer and composite samples showed little variation (all falling within the range of 1–6 MPa), larger differences were reported in ductility. The elongation at breaking point decreased by the incorporation of ceramic nanoparticles, as shown for PCL-based composite fibers [33], [35]. The amount of the mineral phase was likely not sufficient for improving mechanical stability, but it played a direct role in chain polymer entanglements, influencing the ductility properties [25].

Investigating the wettability properties of nanofibrous samples (Table 1), we found that all formulations had a similar hydrophobicity behavior with a contact angle in the range 112–117°, higher than equivalent film samples (69–75°), likely related to the increased surface roughness at the nanoscale. HA and TCP nanoparticles are hydrophilic per se, but their embedding can have no effect on the wettability properties for nanofibrous composite scaffolds [35]. Though a hydrophilic substrate is often better than a hydrophobic one for cell culturing, the latter is often demonstrated able to support a good cell viability, enhancing the biocompatibility and the subsequent cell growth [38], [39].

**Table 1:** Wettability properties of pure polymeric and composite scaffolds, as spin-coated film and NFs.

Scaffold	Contact angle on NFs (°)	Contact angle on film (°)
**PCL**	117±2	75±2
**PCL-HA**	112±10.4	69±1
**PCL-TCP**	116±1	71±1

Results are expressed as mean ± standard deviation. Statistically significant differences (P<0.05) were found for PCL-HA samples.

### Cell adhesion and proliferation

**Figure 4:**
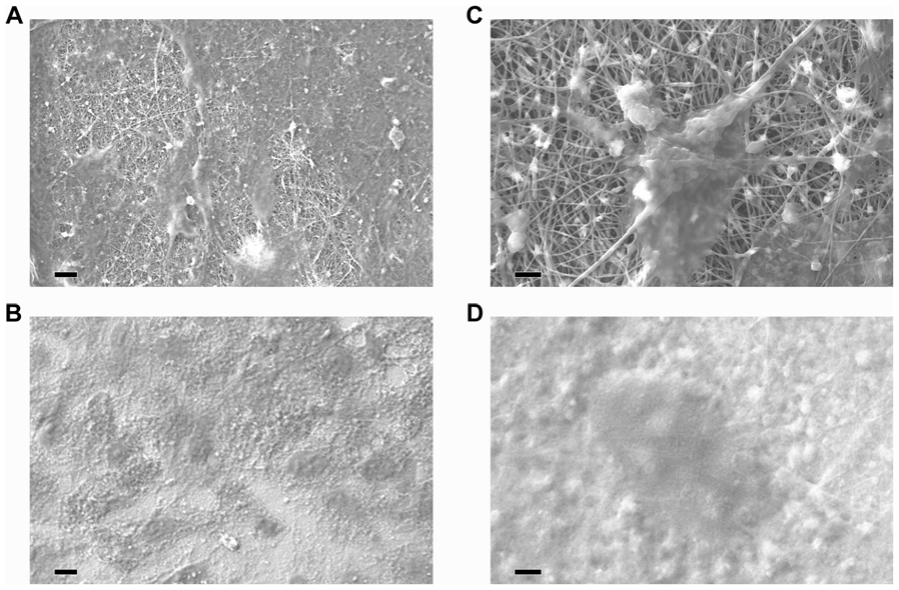
SEM images of hMSCs cultured on nanofibrous scaffolds and films for 7 days. Cell adhesion onto different scaffolds observed by SEM. Cells were able to adhere both onto nanofibrous scaffolds (A, C), and control films (B, D). At higher magnification, a single cell is displayed well attached and spread onto the electrospun NFs, closely associated with the nanofibrous substrate (C). Cells seeded onto control films displayed a less extensive spreading (D). Images from PCL-HA samples, similar images were obtained by PCL and PCL-TCP samples. Bar: 10 µm (A,B) or 2 µm (C,D).

Bone cells are greatly sensitive to the chemical-physical properties of the scaffolds where they are cultured. Surface composition, roughness, and topography all contribute to the osteogenic process, being determinants in cell contact, growth, differentiation and, obviously, cell adhesion represents the initial phase of cell–scaffold communication, triggering numerous cellular responses, including proliferation and differentiation [12], [13], [40], [41]. Here, by loading an equal number of hMSCs onto film or fibrous samples from different materials (PCL, PCL-HA, and PCL-TCP), we observed a good cell adhesion and spreading on all the used scaffold formulations when analyzed by SEM, although in a qualitative way (Fig. 4). However, we could notice that on the nanofibrous mats, cells displayed more interaction with the underlying surface (panel C). Comparable results were provided by optical microscopy analysis (data not shown). Therefore, NFs represent a helpful architecture for allowing cell adhesion. The quantitative in vitro growth of hMSCs, evaluated through the AlamarBlue assay (graphically displayed in Fig. S1), showed a similar trend for all materials, without any statistically significant difference between NFs and controls (films or standard plastic), independently by their chemical composition. These data suggest that the nanostructured topography of these scaffolds, as well as the addition of HA/TCP nanocrystals into the scaffolds, did not affect cell proliferation over time.

### Osteogenic differentiation

**Figure 5:**
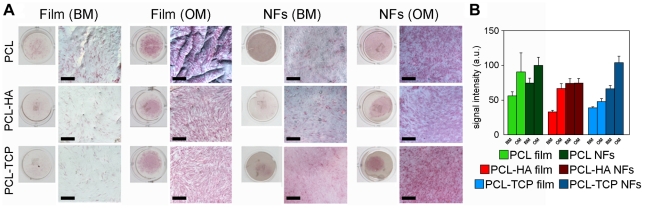
ALP staining. The enzyme activity was evaluated at day 7 of cellular culture onto different samples, either NFs and control films, for each chemical composition, under BM or OM conditions. A) Both a macroscopic view of the Ø 15 mm disks and an optical microscopic image for each sample are shown. Bar: 200 µm. B) Semi-quantification of ALP staining: ALP mean intensity values from image analysis of digital micrographs, after conversion in gray-scale (0–255 bit). Results are reported as mean ± standard deviation.

Following the osteoblastic differentiation model reported by Stein and Lian [42], cells largely proliferate up to 7–14 days and then start to secrete ECM proteins and produce early differentiation markers, such as ALP from day 7. Indeed, ALP is an enzyme belonging to a group of membrane-bound glycoproteins, involved in the pathway resulting in the deposition of minerals on ECM molecules [42]. Therefore, we immuno-histochemically evaluated the activity of the ALP enzyme for hMSCs cultured for one week onto samples either in osteogenic (OM) or in basal growth medium (BM) (Fig. 5). Compared to films, a more evident red staining was observed on nanofibrous scaffolds, with PCL-TCP scaffolds slightly showing a more intensive signal in comparison to PCL and PCL-HA ones (panel A). This result was also confirmed by a semi-quantitative analysis performed comparing the mean ALP signal (panel B). When cells were cultured in OM, ALP enzyme activity was over-expressed, independently by the substrate. Interestingly, cells cultured onto PCL-HA NFs held a fairly positive staining for this enzyme also in BM conditions (see Table S2 for further details).

**Figure 6:**
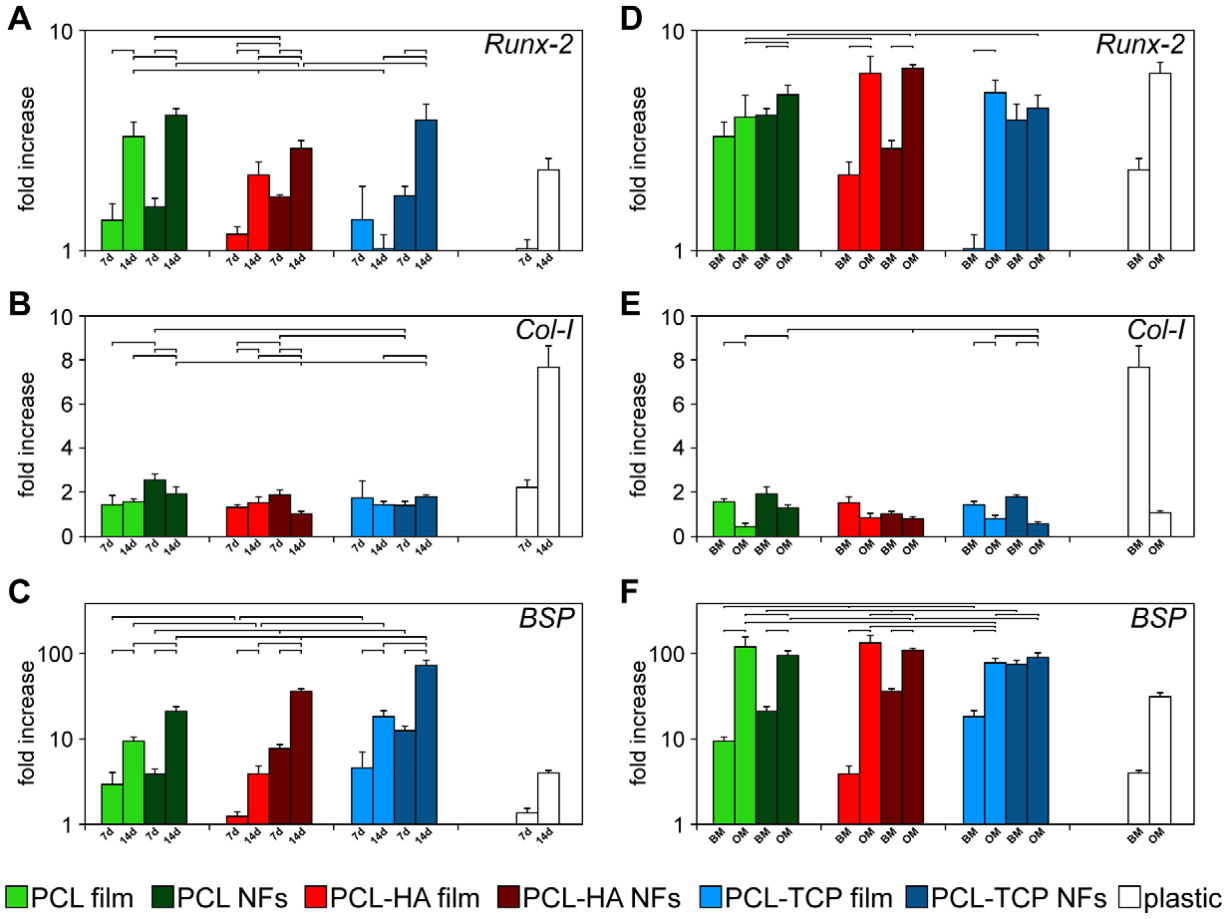
Quantitative real time RT-PCR gene expression analysis of some osteoblast-related genes. A–C) Gene expression levels for hMSCs cultured in basal medium (BM) on different substrates was evaluated over time, comparing results obtained after 7 and 14 days of culture. D–F) The effect of culture medium on gene expression levels is highlighted comparing results obtained at day 14 for all samples cultured either in BM or OM. Expression levels of each gene are reported following normalization to averaged levels previously measured at day 1.

Since ALP can be expressed by other differentiated cells [43], , it is important to study other markers of osteogenic differentiation as well. To this aim, we performed a quantitative analysis of mRNA expression levels, focusing on Runt-related transcription factor 2 (Runx-2), bone sialoprotein (BSP) and type I collagen (Col-1) genes (Fig. 6). Runx2 strongly influences the differentiation process of hMSCs into osteogenesis in the early stage, regulating bone development by G protein-coupled signaling pathway and promoting an up-regulation of ALP, osteopontin, osteocalcin and BSP [45]. Runx-2 expression and activity are usually controlled by external signal, cell-to-cell interaction and growth regulatory factors [46], [47]. Col-1 is fundamental for the development of the bone cell phenotype, being correlated to the formation of the ECM. Col-1 is actively expressed in the first proliferation period and then gradually down-regulated during subsequent osteoblast differentiation, as well as other genes (e.g. transforming growth factor-*β* and fibronectin) [42]. BSP is a highly sulphated and glycosylated phosphoprotein found in bone matrix, considered one of the late markers of mineralized tissue differentiation [48]. Moreover, elevated mRNA expression levels of BSP in vitro are associated with the capacity for bone formation by MSCs [49]. In BM conditions, an almost generalized upregulation of the most typical osteoblast-related genes occurred over time (7 days vs. 14 days, Fig. 6A–C), behavior largely independent by the topographic structure of the scaffolds (NFs vs. films). In particular, type I collagen displayed a roughly homogeneous expression for all the conditions, whereas Runx-2 and BSP were statistically and significantly upregulated over time in all NFs samples. Though the *intrinsic* osteoinducting capability of CaP materials are stated in vivo, the in vitro differentiation of stem cells, cultured on nanostructured scaffolds, is generally evaluated only in osteogenic media, thus suggesting the absence of evident osteoinductive properties relating to the bare scaffold [23], [47], [50]–[52]. Starting from these bases and our above mentioned results, we compared the gene expression levels of cells cultured for two weeks either in basal or osteogenic conditions, in order to evaluate the effect of the culture medium on the osteogenic differentiation (Fig. 6D–F). As expected, we noticed an almost general, statistically significant upregulation of Runx-2 and BSP, when cells were cultured with OM. Collagen type I gene expression levels, on the other side, did not show an up-regulation comparing BM vs. OM culture media. This is consistent with the fact that the expression of Col-I, although essential for osteogenesis during bone formation, is a basic property of functionally different mesenchymal stem cells [53]. Interestingly, only by using PCL-TCP NFs as substrate, both the transcription factor Runx-2 and BSP were equally upregulated in BM as well as in OM. A slight modification in the expression levels of several osteogenic genes, including Runx-2, Col-I and BSP, is reported using a PolyActive™–HA–collagen eletrospun scaffold in basal medium, with no statistically relevant trend of differentiation [26]. Our results suggest an osteogenic commitment of hMSCs cultured on PCL-TCP NFs in basal medium, similar to the results achievable by supplementing growth factors for directing cell fate towards osteoblast lineage.

Our findings indicate the importance of nanotopography and materials in mesenchymal cell populations. While the role of exogenous growth factors as osteogenic commitment regulators is already demonstrated [54], here we report on the likely coupled effect of an ECM-like nanostructure and chemical composition of scaffolds for favoring the differentiation of human stem cells towards bone lineage. Following a biomimetic approach, we have successfully developed an osteoinducting nanostructured substrate, able to mimic a specific physiological microenvironment, finally priming the natural process of cell differentiation, as results from the ALP activity and the expression of important genes in the osteoblast lineage (i.e. Runx-2 and BSP) confirmed. Elucidating the exact mechanism underlying these results will require future studies. These will be focused on examining more in depth the gene expression profile of hMSCs cultured on PCL-TCP nanofibrous meshes, investigating the regulation of other important genes for osteogenesis, such as osteopontin as well as osteocalcin, osteonectin and bone morphogenetic protein-2. Moreover, aiming to the use of this kind of materials as a coating for orthopedic inert implants, we plan to extend the mechanical investigation to more specific tests (e.g. delamination and abrasion tests), and, finally, study the effects of NFs-coated implants on bone regeneration in vivo.

### Conclusion

Here we show that scaffold properties play a pivotal role in controlling the cell growth and impose a direct influence on intracellular responses and cell fate. Cell adhesion, spreading and proliferation represent the initial phase of cell–scaffold communication, which subsequently effect differentiation and mineralization. In this study, the effects of scaffold composition and nanostructured topography were analyzed on cell morphology, growth and osteogenic differentiation of hMSCs in vitro.

The most basic function of an artificial nanostructured ECM is to act as a physical substrate for cell attachment and as three-dimensional microenvironment which serves to organize cells and provides signals for cellular differentiation and maturation. Moreover, the presence of an inorganic cue (i.e. TCP nanocrystals) on the nanofiber surface positively affected differentiation towards an osteogenic commitment. In conclusion, our results indicate that composite nanofibers can be offered as a potential bone regenerative biomaterial for stem cell based therapies.

## Supporting Information

Figure S1 **Cell proliferation, evaluated by AlamarBlue assay.** The vertical bars show standard deviations.
(TIF)

Table S1 **Primer sequences and annealing temperature for real time RT-PCR.**
(DOCX)

Table S2 **Statistical analysis for ALP stained scaffolds.** Statistically significant differences (P<0.05) are marked by the star.
(DOCX)

## References

[1] Bianco P, Robey PG (2001). Stem cells in tissue engineering.. Nature.

[2] Hubbell JA (1995). Biomaterials in tissue engineering.. Nat Biotechnol.

[3] Langer R, Tirrell DA (2004). Designing materials for biology and medicine.. Nature.

[4] Hollister SJ (2005). Porous scaffold design for tissue engineering.. Nat Mater.

[5] Hollister SJ, Maddox RD, Taboas JM (2002). Optimal design and fabrication of scaffolds to mimic tissue properties and satisfy biological constraints.. Biomaterials.

[6] Zhang L, Webster TJ (2009). Nanotechnology and nanomaterials: Promises for improved tissue regeneration.. Nano Today.

[7] Kon E, Muraglia A, Corsi A, Bianco P, Marcacci M (2000). Autologous bone marrow stromal cells loaded onto porous hydroxyapatite ceramic accelerate bone repair in critical-size defects of sheep long bones.. J Biomed Mater Res.

[8] LeGeros RZ (2008). Calcium phosphate-based osteoinductive materials.. Chem Rev.

[9] Sanchez-Salcedo S, Balas F, Izquierdo-Barba I, Vallet-Regi M (2009). In vitro structural changes in porous HA/β-TCP scaffolds in simulated body fluid.. Acta Biomater.

[10] Deng M, Nair LS, Nukavarapu SP, Jiang T, Kanner WA (2010). Dipeptide-based polyphosphazene and polyester blends for bone tissue engineering.. Biomaterials.

[11] Savarino L, Baldini N, Greco M, Capitani O, Pinna S (2007). The performance of poly-ε-caprolactone scaffolds in a rabbit femur model with and without autologous stromal cells and BMP4.. Biomaterials.

[12] Dalby MJ, McCloy D, Robertson M, Wilkinson CD, Oreffo RO (2006). Osteoprogenitor response to defined topographies with nanoscale depths.. Biomaterials.

[13] Tran N, Webster TJ (2009). Nanotechnology for bone materials.. Wiley Interdiscip Rev Nanomed Nanobiotechnol.

[14] Dalby MJ, Gadegaard N, Tare R, Andar A, Riehle MO (2007). The control of human mesenchymal cell differentiation using nanoscale symmetry and disorder.. Nat Mater.

[15] Oh S, Brammer KS, Li YSJ, Teng D, Engler AJ, Chien S (2009). Stem cell fate dictated solely by altered nanotube dimension.. Proc Natl Acad Sci U S A.

[16] Parekh SH, Chatterjee K, Lin-Gibson S, Moore NM, Cicerone MT, Young MF (2011). Modulus-driven differentiation of marrow stromal cells in 3D scaffolds that is independent of myosin-based cytoskeletal tension.. Biomaterials.

[17] Yim EKF, Pang SW, Leong KW (2007). Synthetic nanostructures inducing differentiation of human mesenchymal stem cells into neuronal lineage.. Exp Cell Res.

[18] Tay CY, Pal M, Yu H, Leong WS, Tan NS (2011). Bio-inspired micropatterned platform to steer stem cell differentiation.. Small.

[19] Hynes RO (2009). The extracellular matrix: not just pretty fibrils.. Science.

[20] Tsang KY, Cheung MC, Chan D, Cheah KSE (2010). The developmental roles of the extracellular matrix: beyond structure to regulation.. Cell Tissue Res.

[21] Barnes CP, Sell SA, Boland ED, Simpson DG, Bowlin GL (2007). Nanofiber technology: Designing the next generation of tissue engineering scaffolds.. Adv Drug Deliv Rev.

[22] Xin X, Hussain M, Mao JJ (2007). Continuing differentiation of human mesenchymal stem cells and induced chondrogenic and osteogenic lineages in electrospun PLGA nanofiber scaffold.. Biomaterials.

[23] Hild N, Schneider OD, Mohn D, Luechinger NA, Koehler FM (2011). Two-layer membranes of calcium phosphate/collagen/PLGA nanofibres: in vitro biomineralisation and osteogenic differentiation of human mesenchymal stem cells.. Nanoscale.

[24] Hu J, Feng K, Liu X, Ma PX (2009). Chondrogenic and osteogenic differentiations of human bone marrow-derived mesenchymal stem cells on a nanofibrous scaffold with designed pore network.. Biomaterials.

[25] Thomas V, Dean DR, Jose MV, Mathew B, Chowdhury S (2007). Nanostructured biocomposite scaffolds based on collagen coelectrospun with nanohydroxyapatite.. Biomacrom.

[26] Nandakumar A, Fernandes H, de Boer J, Moroni L, Habibovic P (2010). Fabrication of bioactive composite scaffolds by electrospinning for bone regeneration.. Macromol Bioscience.

[27] Li C, Vepari C, Jin HJ, Kim HJ, Kaplan DL (2006). Electrospun silk-BMP-2 scaffolds for bone tissue engineering.. Biomaterials.

[28] Zhang Y, Venugopal JR, El-Turki A, Ramakrishna S, Su B (2008). Electrospun biomimetic nanocomposite nanofibers of hydroxyapatite/chitosan for bone tissue engineering.. Biomaterials.

[29] Teng SH, Lee EJ, Wang P, Kim HE (2008). Collagen/hydroxyapatite composite nanofibers by electrospinning.. Mater Lett.

[30] Ngiam M, Liao S, Patil AJ, Cheng Z, Chan CK (2009). The fabrication of nano-hydroxyapatite on PLGA and PLGA/collagen nanofibrous composite scaffolds and their effects in osteoblastic behavior for bone tissue engineering.. Bone.

[31] Li WJ, Tuli R, Huang X, Laquerriere P, Tuan RS (2005). Multilineage differentiation of human mesenchymal stem cells in a three-dimensional nanofibrous scaffold.. Biomaterials.

[32] Kim HW (2007). Biomedical nanocomposites of hydroxyapatite/polycaprolactone obtained by surfactant mediation.. J Biomed Mater Res A.

[33] Patlolla A, Collins G, Livingston Arinzeh T (2010). Solvent-dependent properties of electrospun fibrous composites for bone tissue regeneration.. Acta Biomat.

[34] Phipps MC, Clem WC, Catledge SA, Xu Y, Hennessy KM (2011). Mesenchymal stem cell responses to bone-mimetic electrospun matrices composed of polycaprolactone, collagen I and nanoparticulate hydroxyapatite.. PLoS One.

[35] Venugopal JR, Low S, Choon AT, Kumar AB, Ramakrishna S (2008). Nanobioengineered electrospun composite nanofibers and osteoblasts for bone regeneration.. Art Organs.

[36] Binulal NS, Deepthy M, Selvamurugan N, Shalumon KT, Suja S (2010). Role of nanofibrous poly(caprolactone) scaffolds in human mesenchymal stem cell attachment and spreading for in vitro bone tissue engineering-response to osteogenic regulators.. Tissue Eng.

[37] Kolambkar YM, Peister A, Ekaputra AK, Hutmacher DW, Guldberg RE (2010). Colonization and osteogenic differentiation of different stem cell sources on electrospun nanofiber meshes.. Tissue Eng.

[38] Polini A, Pagliara S, Stabile R, Netti GS, Roca L (2010). Collagen-functionalised electrospun polymer fibers for bioengineering applications.. Soft Matter.

[39] Woo KM, Chen VJ, Ma PX (2003). J Biomed Mater Res.

[40] Dalby MJ, Andar A, Nag A, Affrossman S, Tare R (2008). Genomic expression of mesenchymal stem cells to altered nanoscale topographies. R. Soc.. Interface.

[41] Martinez E, Lagunas A, Mills CA, Rodriguez-Segui S, Estevez M (2009). Stem cell differentiation by functionalized micro- and nanostructured surfaces.. Nanomedicine-UK.

[42] Stein GS, Lian JB (1993). Molecular mechanisms mediating proliferation/differentiation interrelationships during progressive development of the osteoblast phenotype.. Endocrine Rev.

[43] Hinnebusch BF, Siddique A, Henderson JW, Malo MS, Zhang W (2004). Enterocyte differentiation marker intestinal alkaline phosphatase is a target gene of the gut-enriched Kruppel-like factor.. Am J Physiol Gastrointest Liver Physiol.

[44] Matsumoto H, Erickson RH, Gum JR, Yoshioka M, Gum E (1990). Biosynthesis of alkaline phosphatase during differentiation of the human colon cancer cell line Caco-2.. Gastroenterology.

[45] Teplyuk NM, Galindo M, Teplyuk VI, Pratap J, Young DW (2008). Runx2 regulates G protein-coupled signaling pathways to control growth of osteoblast progenitors.. J Biological Chemistry.

[46] Teixeira AI, McKie GA, Foley JD, Bertics PJ, Nealey PF (2006). The effect of environmental factors on the response of human corneal epithelial cells to nanoscale substrate topography.. Biomaterials.

[47] Kim HJ, Kim JH, Bae SC, Choi JH, Kim HJ (2003). The protein kinase C pathway plays a central role in the fibroblast growth factor-stimulated expression and transactivation activity of Runx2.. J Biological Chemistry.

[48] Franceschi RT (1999). The developmental control of osteoblast-specific gene expression: role of specific transcription factors and the extracellular matrix environment.. Crit Rev Oral Biol Med.

[49] Satomura K, Krebsbach P, Bianco P, Gehron Robey P (2000). Osteogenic imprinting upstream of marrow stromal cell differentiation.. J Cell Biochem.

[50] Kaur G, Valarmathi MT, Potts JD, Jabbari E, Sabo-Attwood T (2010). Regulation of osteogenic differentiation of rat bone marrow stromal cells on 2D nanorod substrates.. Biomaterials.

[51] You MH, Kwak MK, Kim DH, Kim K, Lechenko A (2010). Synergistically enhanced osteogenic differentiation of human mesenchymal stem cells by culture on nanostructured surfaces with induction media.. Biomacromolecules.

[52] Ruckh TT, Kumar K, Kipper MJ, Popat KC (2010). Osteogenic differentiation of bone marrow stromal cells on poly(ε-caprolactone) nanofiber scaffolds.. Acta Biomat.

[53] Frank O, Heim M, Jakob M, Barbero A, Schäfer (2002). Real-time quantitative RT-PCR analysis of human bone marrow stromal cells during osteogenic differentiation in vitro.. J Cell Biochem.

[54] Muraglia A, Martin I, Cancedda R, Quarto R (1998). A nude mouse model for human bone formation in unloaded conditions.. Bone.

