# Supplementary figures and images for: Osteoinduction of Human Mesenchymal Stem Cells by Bioactive Composite Scaffolds without Supplemental Osteogenic Growth Factors
Source: PLoS One. 2011 Oct 12;6(10):e26211. doi: 10.1371/journal.pone.0026211 (PMC3192176; doi:10.1371/journal.pone.0026211)

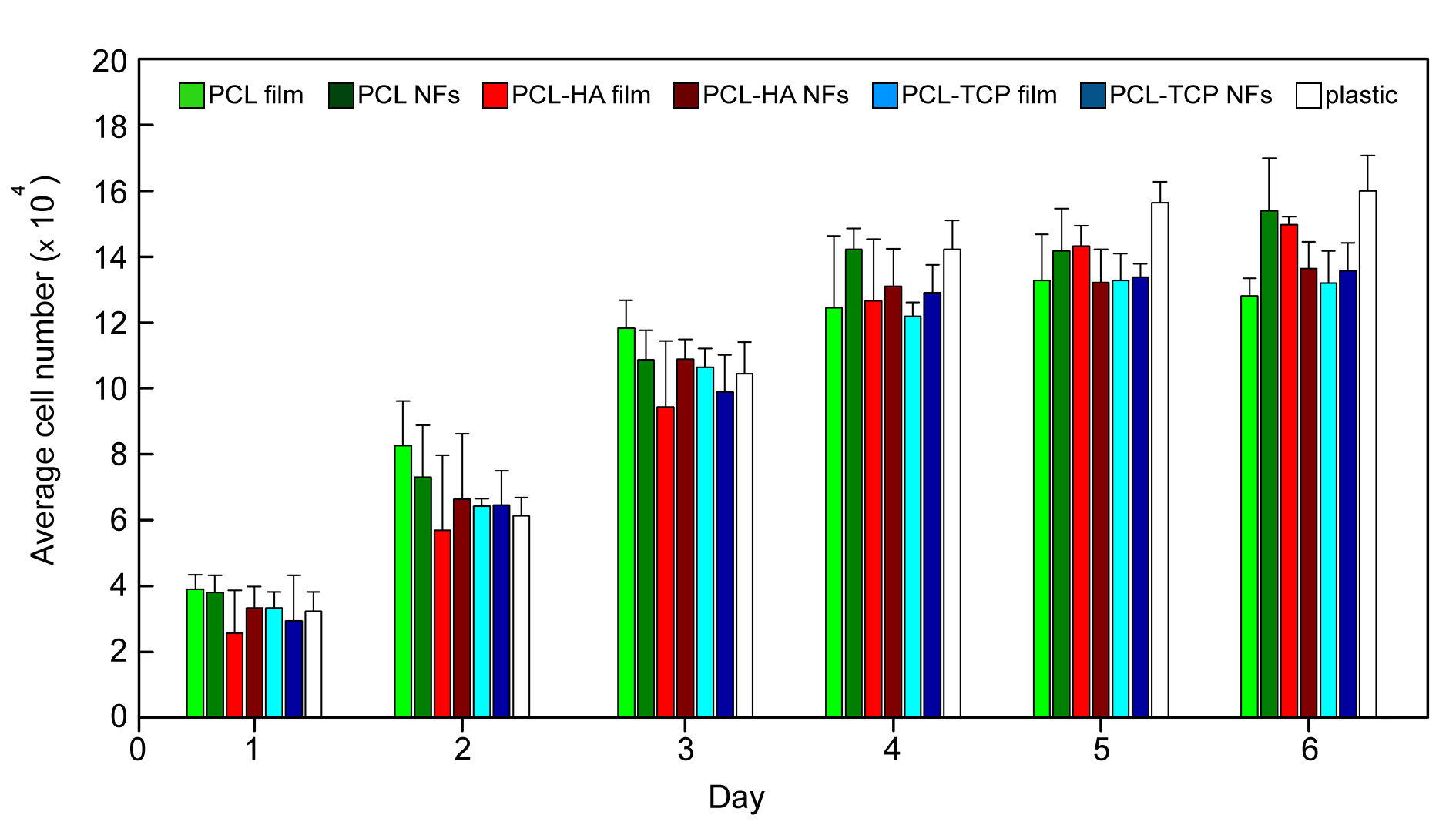

Supplement: Figure S1 — Cell proliferation, evaluated by AlamarBlue assay. The vertical bars show standard deviations. (TIF) [file pone.0026211.s001.tif]
