# Supplementary material for: Osteoinduction of Human Mesenchymal Stem Cells by Bioactive Composite Scaffolds without Supplemental Osteogenic Growth Factors
Source: PLoS One. 2011 Oct 12;6(10):e26211. doi: 10.1371/journal.pone.0026211 (PMC3192176; doi:10.1371/journal.pone.0026211)
Supplement: Table S1 — Primer sequences and annealing temperature for real time RT-PCR. (DOCX) [file pone.0026211.s002.docx]

|  | **Primer forward** | **Primer reverse** | **Tm** |
| --- | --- | --- | --- |
| **GAPDH** | 5’-ACC CAC TCC TCC ACC TTT GA-3’ | 5’-CTG TTG CTG TAG CCA AAT TCG T-3’ | 61°C |
| **RUNX-2** | 5’-CTT CAT TCG CCT CAC AAA CA-3’ | 5’-TTG ATG CCA TAG TCC CTC CT-3’ | 58°C |
| **Col-I** | 5’-TTG CTC CCC AGC TGT CTT AT-3’ | 5’-TCC CCA TCA TCT CCA TTC TT-3’ | 58°C |
| **BSP** | 5’-GAA GAA GAG GAG ACT TCA AAT G-3’ | 5’-TAT CCC CAG CCT TCT TGG GA-3’ | 61°C |

Table S1. Primer sequences and annealing temperature for real time RT-PCR.
