# Supplementary material for: Osteoinduction of Human Mesenchymal Stem Cells by Bioactive Composite Scaffolds without Supplemental Osteogenic Growth Factors
Source: PLoS One. 2011 Oct 12;6(10):e26211. doi: 10.1371/journal.pone.0026211 (PMC3192176; doi:10.1371/journal.pone.0026211)
Supplement: Table S2 — Statistical analysis for ALP stained scaffolds. Statistically significant differences (P<0.05) are marked by the star. (DOCX) [file pone.0026211.s003.docx]

| **PCL film BM** |  |  |  |  |  |  |  |  |  |  |  |  |
| --- | --- | --- | --- | --- | --- | --- | --- | --- | --- | --- | --- | --- |
| **PCL film OM** | * |  |  |  |  |  |  |  |  |  |  |  |
| **PCL NFs BM** | * | * |  |  |  |  |  |  |  |  |  |  |
| **PCL NFs OM** | * | * | * |  |  |  |  |  |  |  |  |  |
| **PCL-HA film BM** | * | * | * | * |  |  |  |  |  |  |  |  |
| **PCL-HA film OM** | * | * | * | * | * |  |  |  |  |  |  |  |
| **PCL-HA NFs BM** | * | * |  | * | * | * |  |  |  |  |  |  |
| **PCL-HA NFs OM** | * | * |  | * | * | * |  |  |  |  |  |  |
| **PCL-TCP film BM** | * | * | * | * |  | * | * | * |  |  |  |  |
| **PCL-TCP film OM** | * | * | * | * | * | * | * | * | * |  |  | |
| **PCL-TCP NFs BM** | * | * | * | * | * |  | * | * | * | * |  |  |
| **PCL-TCP NFs OM** | * | * | * |  | * | * | * | * | * | * | * |  |
| **Scaffold** | **PCL film BM** | **PCL film OM** | **PCL NFs BM** | **PCL NFs OM** | **PCL-HA film BM** | **PCL-HA film OM** | **PCL-HA NFs BM** | **PCL-HA NFs OM** | **PCL-TCP film BM** | **PCL-TCP film OM** | **PCL-TCP NFs BM** | **PCL-TCP NFs OM** |

**Table. S2. Statistical analysis for ALP stained scaffolds.** Statistically significant differences (P < 0.05) are marked by the star.
